# Patient-reported outcome, clinician-reported outcome, and patient satisfaction with treatment by crisis resolution teams: a multicenter pre-post study of outcome and associated factors in Norway

**DOI:** 10.1186/s12888-024-05543-3

**Published:** 2024-01-31

**Authors:** T. Ruud, N. Hasselberg, J. Siqveland, K. H. Holgersen

**Affiliations:** 1 Division of Mental Health Services, Akershus University Hospital, Lørenskog, Norway; 2 Institute of Clinical Medicine, University of Oslo, Oslo, Norway; 3 National Center for Suicide Research and Prevention, University of Oslo, Oslo, Norway; 4 Nidelv Community Mental Health Centre, Tiller, Department of Mental Health, St. Olavs Hospital, Trondheim, Norway; 5 Department of Psychology, Norwegian University of Science and Technology, Trondheim, Norway

**Keywords:** Crisis resolution teams, Patient-reported outcome, Clinician-reported outcome, Patient satisfaction, Predictors of outcome

## Abstract

**Background:**

Crisis resolution teams (CRTs) have become a part of mental health services in many high-income countries. Many studies have investigated the impact of CRTs on acute admissions to inpatient units, but very few studies have investigated patient-reported and clinician-reported outcomes for CRT service users. Our aims were to study patient-reported and clinician-reported outcomes of CRT treatment, how the outcomes were associated with characteristics of the service user and the treatment, and whether outcomes were different across CRTs.

**Methods:**

The study was a pre-post observational multicenter study of 475 patients receiving treatment from 25 CRTs in urban and rural areas in Norway. There was no control group. Outcomes were change in mental health status reported by service users using CORE-10 and by clinicians using HoNOS. Patient satisfaction was measured using CSQ-8 at the end of the treatment. Components of CRT accessibility and interventions were measured by clinicians reporting details on each session with the service user. CRT model fidelity was measured using the CORE CRT Fidelity Scale version 2. We used paired t-tests to analyze outcomes and linear mixed modeling to analyze associations of the outcomes with the characteristics of service users and the treatment provided. Using independent t-tests, we analyzed differences in outcomes and patient satisfaction between two clusters of CRTs with differences in accessibility.

**Results:**

The patient-reported outcomes and the clinician-reported outcomes were significantly positive and with a large effect size. Both were significantly positively associated with practical support and medication management and negatively associated with collaboration with mental health inpatient units. Patient satisfaction was high at the end of the treatment. CRTs with higher accessibility had a significantly better clinician-reported outcome, but no significant differences were reported for patient-reported outcomes or patient satisfaction.

**Conclusions:**

CRT treatment led to improved symptom status as reported by patients and clinicians, as well as high patient satisfaction. Practical support and medication management were the interventions most strongly associated with positive outcomes. Some of the variations in outcomes were at the team level. Patient- and clinician-reported outcomes should be used more in studies on the effect of treatment provided by crisis resolution teams.

**Supplementary Information:**

The online version contains supplementary material available at 10.1186/s12888-024-05543-3.

## Background

Crisis resolution teams (CRTs) are intended to provide rapid assessment in mental health crises and to offer intensive home treatment as an alternative to acute admission, if feasible [1]. The teams aim to reduce the use of inpatient stays to manage crises and provide less restrictive treatment compared to inpatient admissions [1]. CRTs are now a part of mental health services in many high-income countries.

Many studies have investigated the impact of CRTs on acute admissions to inpatient units and patient satisfaction with CRT care, but very few studies have investigated patient and clinician-reported mental health and symptoms outcomes for treatment in CRTs. A recent scoping review of studies of CRTs found that only 38 of 129 included studies were on treatment effects [2], mostly investigating reduction in admissions to acute psychiatric inpatient units as outcomes. Earlier reviews of studies comparing CRTs to treatment as usual have reported similar outcomes in mental health, functioning, and quality of life. This suggests that CRTs deliver results on par with treatment in acute inpatient units for most patients [3, 4]. A 2015 Cochrane review concluded that crisis intervention appears to improve mental state (two studies, low-quality evidence) and that after crisis intervention, patients were more satisfied with their care (one study, moderate quality evidence) [5]. Two recent randomized controlled trials on CRT treatment compared to acute inpatient treatment also found a reduction in the use of inpatient treatment but no significant group differences in clinical outcome and patient satisfaction [6, 7]. The recent cluster-randomized study by the research team that developed the CORE CRT Fidelity Scale found no significant differences in patient satisfaction between the intervention group of CRTs with significant higher fidelity and the control group [8, 9]. An earlier pre-post study of CRTs in Norway found moderate improvement in mental health [10]. Two recent pre-post studies of CRT patients in Spain also found significant improvement in psychiatric problems [11, 12], and a recent prospective observational study of CRT patients in Greece found improvement in clinician-rated psychiatric problems pre-post and compared to treatment as usual [13]. While some studies indicate that CRT home treatment is not associated with an increased risk of violence or suicide, an extensive recent overview of various approaches in acute psychiatric care states that ‘crisis assessment at home is not suitable when someone requires urgent medical tests or treatment (for example, following an overdose or other self‐harm)’ [14].

There have been many studies on the implementation and components of the CRT model. However, the few studies on patient-reported or clinician-reported clinical outcomes have, to a limited extent, explored how the clinical outcomes are associated with patient characteristics, characteristics of the situation or mental crisis, accessibility to the crisis treatment, or the various interventions provided by the CRTs [2, 3, 15]. A recent rapid review found that few studies explored the actual content of care offered by CRTs and the effect of specific interventions on outcomes [16]. Another review concluded that there is little evidence for the impact of CRTs on clinical outcomes such as symptom reduction or relapse [15]. The authors also commented that testing the effect on various outcomes of each component of CRT as a complex intervention would demand many randomized controlled trials. They suggested that a potential alternative would be to study service characteristics and interventions delivered across large numbers of teams, investigating outcomes at the patient level using multilevel modeling [15]. Such multilevel modeling studies could analyze fidelity scores for various treatment components at team level [15]. Another option could be to include measurements of the various treatment components as they are provided to individual service users. There has been little research on which interventions CRTs actually use, how they are implemented, and their effect [17]. A recent study has reported a way to measure the delivery of specific components of accessibility and interventions in CRT’s crisis treatment [18]. To summarize, there is a need for more knowledge about how different interventions and practices by CRTs affect patient care and outcomes.

## Methods

### Aims

The aims of the current study were to study (a) patient satisfaction and patient- and clinician-reported outcomes of CRT treatment; (b) the association between characteristics of the crisis and the service users, treatment accessibility, patient satisfaction, and treatment outcomes; and (c) differences in outcomes and patient satisfaction across teams.

Our hypotheses were: (a) patient-reported outcome and clinician-reported outcome and patient satisfaction were significantly positive; (b) patient- and clinician-reported outcomes and patient satisfaction were significantly associated with severity of mental problems and crisis at admission, with access to and intensity of the treatment, and with interventions provided; and (c) teams with higher adherence to key elements of the CRT model had significantly better outcomes.

### Context

Norwegian mental health services are mostly public services provided by the 19 health trusts, which also provide general hospital services and other specialized health services [19]. The mental health and substance abuse services for adults in each health trust consist of hospital inpatient units and two or more community mental health centers (CMHCs) serving local catchment areas. The CMHCs have outpatient clinics, CRTs, other mobile teams, and local inpatient units [20]. The CMHCs collaborate with general practitioners (GPs), primary health and social care in the municipalities, and the hospital units.

### Design

The study was a pre-post observational multicenter study with no control group. The study was conducted by the Department of Research and Development, Division of Mental Health Services, Akershus University Hospital, in collaboration with the national Network for Acute Psychiatric Services. The project group included researchers (psychiatrist, psychologists) and experienced mental health service user experts.

According to CRT models and guidelines, we defined the maximum time for crisis treatment to be eight weeks. For service users who were not discharged from CRT treatment within eight weeks, the discharge form and patient questionnaire were completed at eight weeks to have comparable data across teams and service users.

In this article, we followed the STROBE statement for reporting observational studies, version 4 for cohort studies [21, 22].

### Recruitment and preparations of the crisis resolution teams

The CRTs were recruited by an invitation sent in September 2014 to all 19 health trusts in Norway. The CRTs participated in discussions of the measures and practical procedures by email, in the two-day network meeting of the Acute Network, and in a workshop during the final preparations. Final forms and questionnaires with information and instructions were distributed in February 2015, and the patient inclusion and data collection started in March 2015. The CRTs collected the data in 2015–2016 as a part of their clinical work during the project period. We aimed to recruit 30 CRTs based on the interests shown from CRTs in the national Network for Acute Psychiatric Services. Thirty CRTs registered for participation in the study. However, due to organizational changes, two of these teams withdrew their participation before the study started.

Power calculation of minimum sample size: We chose clinical change in Clinical Outcome in Routine Evaluation 10 (CORE-10) as the primary outcome measure in our study. In a sample of 321 GP patients, the mean (SD) of CORE-10 was 20.2 (7.9), and reliable clinical change in CORE-10 is estimated to be a score reduction of 6 points [23]. To find a significant pre-post change of 6 points or more measured with 5% two-tailed significance and power for 90%, we estimated that we needed at least a total of 37 participants across CRTs. However, we wanted a much larger total sample to include 20 or more explanatory variables (service user characteristics, accessibility variables, and interventions) and to measure the amount of total variance found on the team level (differences between teams) in regression analyses using linear mixed models. From the number that CRTs in the network expected to recruit and from our experiences from an earlier multi-center study of CRTs in Norway with 680 participants from eight CRTs [10], we decided to encourage each CRT to recruit 40 service users (also including some room for variance in recruitment and dropouts).

### Measures

#### Outcome measures

The primary outcome was change from the beginning to the end of treatment (pre-value minus post-value) on patient-reported outcome using CORE-10 [24, 25]. CORE-10 is a brief questionnaire that has a five-point response scale (0 “not at all” to 4 “most or all the time”), adequate psychometric properties, and availability in Norwegian. Total sum score is from 0 to 40 and total mean score is from 0 to 4, with higher scores for more serious symptoms. CORE-10 consists of ten questions from Clinical Outcomes in Routine Evaluation-Outcome Measure (CORE-OM), and the Norwegian version of CORE-OM has been shown to have excellent psychometric properties [26]. CORE-10 has questions that are relevant to the dimensions of mental crisis regarding depression, anxiety, trauma, suicidal risk, functioning, and support. Questions 2 and 3 are positively formulated; they are reversed when calculating the total mean of CORE-10. The internal consistency (Cronbach’s alpha) of CORE-10 for our sample was 0.75 at the start and 0.88 at the end of the treatment.

The secondary outcome was change in clinician-reported outcome measures from the beginning to the end of treatment (pre-value minus post-value) using the Health of the Nation Outcome Scales (HoNOS) [27]. HoNOS has 12 items with a five-point response scale (0 “no problem” to 4 “serious or very serious problem”). Total sum score is from 0 to 48 and total mean score is from 0 to 4, with higher scores for more serious problems. It is used as a routine clinical measure in mental health services in many countries, including by CRTs [28]. It has good psychometric properties [29]. It has been used in several studies on CRTs in the UK and Norway [10, 30, 31]. The CRT team members were trained in rating HoNOS through e-learning based on the training on rating HoNOS in the UK.

Another secondary outcome measure was service user satisfaction measured using Client Satisfaction Questionnaire 8 (CSQ-8) at the end of treatment [32]. Each item has a four-point response scale (1 to 4) with higher score for higher satisfaction, a total sum score of 8–32, and each question has a specific text for each point. The internal consistency (Cronbach’s alpha) of CSQ-8 for our sample was 0.90 at the end of the treatment.

#### Independent variables

Data on age, gender, whether the service user was living alone, previously known mental illness, diagnosis of psychosis or not, earlier contact with the team, and the referral process and start of the treatment were collected by the CRT on a registration form. Perceived social support from family and network was measured using the Crisis Support Scale (CSS) completed by the service users at the start and the end of the treatment [33]. The questionnaire has seven items with a seven-point response scale (1 “never” to 7 “always”), a mean score 1 to 7, and higher scores for more support. A review of the psychometric properties of CSS in 11 trauma studies concluded that the scale appears to be very robust, with good reliability and discriminatory power, and adequate internal consistency [34]. The internal consistency (Cronbach’s alpha) of CSS for our sample was 0.69 at the end of the treatment.

The service users’ experiences of the crisis at the start of treatment were measured using the Crisis State Assessment Scale (CSAS) questionnaire with ten items [35]. The scale for each item was reduced from seven to five points to fit with other measures in the questionnaire, and the wording of the five steps was rephrased from expressions of how often to expressions of to what extent (1 “not at all” to 5 “to a very large extent”) to focus on the experience of the crisis. In the current study’s exploratory factor analysis (principal component analysis with varimax rotation and Kaiser’s criteria of eigenvalues at 1 or more), we identified two factors in line with the original study: “Crisis experience” (4 items, mean score from 1 to 5, higher score for more serious crisis) and “Not coping with crisis” (6 items, mean score from 1 to 5, higher score for lower coping). Using the mean of these two factors as subscales, the internal consistency (Cronbach’s alpha) of these subscales for our sample at the start of the treatment was 0.88 and 0.81, respectively.

The response time to the first session with the CRT was calculated as the time interval from the date and time for the received referral to the first session. Additionally, information recorded was whether the service user was seen on the referral day, whether the service user was self-referred, length of treatment (in weeks), and how the treatment ended.

Data on access to the CRT treatment (date, time of the day, location of session, and duration of session) were recorded on a session registration form filled in by the clinician immediately following each session. Based on these data, we calculated treatment intensity (sessions per week), duration of sessions (ordinal scale as shown in Table 1), proportion of sessions outside the team’s location, and proportion of sessions outside ordinary working hours. The clinician also rated 26 possible session activities regarding assessment, treatment, and collaboration. Each activity was rated on a four-point scale if present (1 = little, 2 = some, 3 = much, 4 = very much) and counted as 0 (not done) if not rated. Factor analysis of the treatment activities resulted in four factors: practical support, psychological interventions, family involvement, and medication management. Factor analysis of collaboration activities resulted in one factor on collaboration with mental health inpatient units (mostly CRTs preparing and implementing inpatient admissions) and one on collaboration with general practitioners and primary care (CRTs cooperating with GPs and municipal primary care teams). The variables and analyses from the session registration form are reported in detail in a previous paper on CRT accessibility and interventions in Norway [18].

**Table 1:** Characteristics of service users (*N* = 475), situation at the start of treatment, and crisis treatment provided to the service users

**Categorical variables**			**N**	**%**
**Age group**				
Under 20 years			23	4.8
20–29 years			145	30.5
30–39 years			105	22.1
40–49 years			85	17.9
50–59 years			66	13.9
60–69 years			38	8.0
70–79 years			9	1.9
80–89 years			4	0.8
**Sex**				
Male			189	39.8
Female			286	60.2
**Living alone**				
No			317	66.7
Yes			158	33.3
**Previously known mental illness**				
No			317	66.7
Yes			158	33.3
**Psychosis**				
No			437	92.0
Yes			38	8.0
**Earlier contact with crisis team**				
No			361	76.0
Yes			114	24.0
**Crisis how acute**				
Sudden event			58	12.2
Developed quickly			70	14.7
Gradually development			147	30.9
Has lasted for a long time			200	42.1
**Crisis duration**				
Last 24 h			7	1.5
Last days			46	9.7
Last 1–2 weeks			89	18.7
Several weeks			333	70.1
**Self-referral (direct contact)**				
No			386	81.3
Yes			89	18.7
**Time to first meeting after referral**				
Same day			186	39.2
Next day			145	30.5
2–3 days			75	15.8
4–7 days			51	10.7
More than a week			18	3.8
**Seen on referral day**				
No			289	60.8
Yes			186	39.2
**Continuous variables**			**Mean**	**SD**
**Problems at the start of treatment**				
Patient-reported symptoms CORE10			2.44	0.61
Clinician-reported problems HONOS			0.93	0.40
Crisis Support Scale			4.25	0.97
Crisis experience (CSAS subscale)			4.39	0.71
Not coping with crisis (CSAS subscale)			3.72	0.78
**Characteristics of crisis treatment**				
Treatment length (weeks)			3.05	2.64
Treatment intensity (sessions pr week)			1.56	0.71
Average duration of sessions (ordinal scale)			3.05	0.53
Ordinal scale	Minutes	N (%)		
1	05–20	83 ( 4.7%)		
2	25–40	221 (12.6%)		
3	45–60	1089 (62.2%)		
4	65–90	353 (20.2%)		
5	90 or more	4 ( 0.2%)		
Proportions of sessions outside team’s location			0.38	0.43
Proportions of sessions outside working hours			0.20	0.30
**Proportion with various interventions**				
Practical support			0.08	0.19
Psychological interventions			0.90	0.21
Family involvement			0.12	0.24
Medication management			0.11	0.23
Collaboration with mental health inpatient units			0.05	0.17
Collaboration with GPs and primary care			0.14	0.26
**CORE CRT Fidelity Scale**				
Fidelity total mean (range 1 to 5)			2.75	0.29
Fidelity total score (range 39 to 195)			107	11

The CRTs’ fidelity to an evidence-based model of CRTs was measured at team level using the CORE Crisis Resolution Team Fidelity Scale version 2 [8]. The scale has 39 items (total score from 39 to 195, mean score from 1 to 5) and measures key elements such as opening hours, response time, treatment intensity, home treatment, and gatekeeping of acute beds. A mean score of 4.00 and above (total score 156 and above) is considered high fidelity, a mean score of 3.00 to 3.99 (total score 117 to 155) is considered moderate fidelity, and a mean score of lower than 3.00 (lower than total score 117) is considered low fidelity [36]. Details of the fidelity of the CRTs in the current study have been published in a previous paper [36].

### Crisis treatment provided by the crisis resolution teams

The CRTs in the study are described in two previous papers [18, 36]. The CRTs were located at CMHCs with outpatient clinics and, for the most part, with CMHC inpatient units. The populations in the catchment areas ranged from 40,000 to 130,000. The staffing for a CRT averaged 10.0 full-time equivalents (range 4.0–20.4). The average full-time equivalents of the major professional groups in a CRT were 5.4 mental health nurses/nurses, 1.5 clinical psychologists, 1.3 psychiatrists/physicians specializing in psychiatry, and 1.0 social workers. No Norwegian team operated 24/7. However, half of the teams operated extended hours on weekdays, and six of these also had some hours on weekends. The remaining teams operated only during office hours on weekdays. The fidelity total score was from low to moderate, with a moderate variation across the teams [36]. Ratings were high on comprehensive assessment, psychological interventions, visit length, service users’ choice of location, and type of support. Ratings were low on opening hours, gatekeeping acute psychiatric beds, facilitating early hospital discharge, intensity of contact, providing medication, and providing practical support.

### Recruitment of service users and data collection

In the first session, the CRT clinicians asked service users if they wanted to participate in the study. Each team invited potential participants to give informed written consent to participate in the study until the recommended 40 participants had been included.

The CRT team members collected data as part of their clinical work and contact with the service users. Each service user was asked to fill in a questionnaire at the start and end of treatment, and the clinicians filled in a form with information about the service user and their assessment as clinicians at the start and end of treatment. Team member registered data on the CRT treatment delivery to the individual service user on the session registration form after each session. Completed questionnaires and forms were sent to the research group for registration in the electronic database on a research server at Akershus University Hospital.

Three persons from an assessment team, including an experienced service user expert, did fidelity assessments. The assessment team was trained in using the CORE CRT Fidelity Scale version 2. Each CRT was assessed during the period when they included study participants.

### Participants

The only inclusion criterion was that the service user was able to answer the questionnaires in Norwegian or English. Three of the 28 CRTs did not recruit any service users to the study, due to various reasons. Altogether, 1040 service users from 25 CRTs gave written informed consent to participate. The service users’ questionnaire and the clinicians’ registration form were completed at the start and the end of treatment for 645 service users. Of these, 170 (26.6%) had missing data on one or more of the variables for the planned analyses. Therefore, 475 service users are included in the data analyses. There were no significant differences between the sample of 475 services users included in the analyses and the 170 excluded for baseline sex, age group, CORE-10 or HoNOS. Comparing the sample of 475 to the rest of the 1040 giving consent to participate, the were no significant differences for baseline sex, age group, and CORE-10. However, HoNOS total sum at baseline was significantly lower for the sample than for the rest of whose giving consent (11.16 vs 11.90, p 0.022). The median number of service users in the data analyses was 18 per team (range 1–36). Table 1 shows baseline characteristics of the 475 service users, the referral process, the treatment provided, and fidelity of the CRTs.

### Data analyses

The patient-reported outcomes (CORE-10) and clinician-reported outcomes (HoNOS) were analyzed using paired t-tests and calculations of effect sizes (Cohen’s d) for each item and the total sum [37]. For patient-reported outcomes, we also reported the distribution on three levels of severity of psychiatric symptoms based on empirical data of cut-off points of the CORE-10 total sum score reported in the CORE-10 user manual [23]. The three levels were low symptom level (0–10), mild to moderate symptom level (11–25), and serious symptom level (26–40).

We analyzed the association between the independent variables and each of the outcomes using linear mixed models to correctly adjust the estimates for within-team correlations. Empty linear mixed models were used to assess the degree of clustering through an intraclass correlation coefficient (ICC) on team-level. For each of the outcomes we estimated three multiple linear mixed models: Model A, with variables on the situation at the treatment start; Model B, with variables on the treatment provided by the CRT; and Model AB, with variables on both the treatment start and the treatment provided.

We included 13 independent variables in Model A: Three socio-demographic variables (age, sex, living alone); five variables on baseline status (diagnosis of psychosis, previously known mental illness, previous contact with the CRT, severity of patient-reported mental symptoms, severity of clinician-reported problems); and five variables reported by the service users on the current situation (how suddenly the crisis developed, how long the crisis had lasted, experience of the crisis severity, experienced lack of coping with the crisis, experienced crisis support from family and friends).

We included 16 independent treatment variables in Model B: Eight variables on access to the CRT (self-referral, response time, proportion with the first session on referral day, proportion of sessions outside the CRT location, proportion of sessions outside office hours, average duration of sessions, duration of the crisis treatment in weeks, intensity of the crisis treatment as sessions per week); six variables on intervention content (practical support, psychological interventions, family involvement, medication management, collaboration with inpatient services, collaboration with GPs and primary care); and fidelity total mean of the CRT serving the patient. Patient satisfaction at the end of the treatment was also included as an independent variable for the patient-reported and clinician-reported outcomes.

Model AB included the 29 independent variables in Models A and B.

In the linear mixed models, all independent variables were included as fixed effects, while total fidelity was included as random effects with unstructured covariance. Variations of each model were created and tested in a stepwise procedure, eliminating independent variables one by one based on the lowest z-value and highest p-value. For each model, we present the results with the lowest Bayesian Information Criterion (BIC) value.

For readers interested in seeing the results of the complete models of the outcomes with all the independent variables (before the described reduction of variables), we have made this available in supplementary tables in the online Supplementary material.

In the linear mixed models, we also estimated the team-level proportion (intraclass correlation coefficient, ICC) of the total variance of the dependent variable. There are no established guidelines for what should be considered high or low team-level proportion (ICC) of total variance, as this will depend on many factors. However, for these data, we defined high ICC as 25.0% and above, medium ICC as 10.0 – 24.9%, and low ICC as below 10.0%, as in our previous article on measurement of accessibility and interventions in the CRT treatment [18].

Using independent t-tests, we analyzed differences in outcomes and patient satisfaction between two clusters of CRTs identified in a previous paper with a cluster analysis based on three key elements of accessibility (proportion with first session on referral day, proportion of sessions outside CRT location, proportion of sessions outside office hours) [18]. We excluded six teams with less than ten service users in the data analyses. One cluster A with ten teams (271 patients, median 31 patients, range 12–36) had significantly higher accessibility for the three key accessibility elements than the other cluster B with nine teams (182 patients, median 21, range 10–32) [18].

## Results

### Outcomes and service user satisfaction

Table 2 shows the patient-reported outcome, clinician-reported outcome, and patient satisfaction at the treatment end. There was a positive significant pre-post change in the patient-reported outcomes (CORE-10) for the total sum and all items. The effect size for the total sum was large, and the effect size for the single items was small to large. As shown in the second section of Table 2, the distribution of service users on the three defined levels of symptom severity (low, mild to moderate, and serious) had a shift toward lower levels of severity at the end of treatment compared to the start of treatment.

**Table 2:** Patient-rated and clinician-rated outcomes pre-post (*N* = 475). Paired t-tests and effect sizes (Cohen’s d). Descriptive statistics of patient satisfaction at the end of treatment

**Clinical outcome**	**Start of treatment**	**End of treatment**	**Change from start of treatment to end of treatment**		
**Patient-reported outcome**					
** Psychiatric symptoms (CORE-10)**	**Mean (SD)**	**Mean (SD)**	**Mean (SD)**	***P***	**Cohen’s d**
1. Felt tense, anxious or nervous	3.26 (0.81)	2.54 (1.04)	0.72 (1.05)	** < .001**	0.68
2. Felt have someone to turn to for support	2.37 (1.13)	2.68 (1.02)	-0.31 (1.28)	** < .001**	-0.24
3. Felt able to cope when things go wrong	1.77 (0.93)	2.20 (0.92)	-0.43 (1.14)	** < .001**	-0.37
4. Talking to people have felt too much	2.16 (1.13)	1.75 (1.13)	0.41 (1.26)	** < .001**	0.32
5. Felt panic or terror	2.51 (1.16)	1.80 (1.21)	0.72 (1.17)	** < .001**	0.61
6. Made plans to end my life	1.30 (1.34)	0.73 (1.08)	0.58 (1.17)	** < .001**	0.49
7. Had difficulty getting to sleep or staying asleep	2.85 (1.17)	2.23 (1.26)	0.62 (1.28)	** < .001**	0.48
8. Felt despairing or hopeless	2.95 (0.98)	1.96 (1.22)	0.99 (1.17)	** < .001**	0.85
9. Felt unhappy	3.16 (0.94)	2.29 (1.24)	0.87 (1.20)	** < .001**	0.72
10. Distressing unwanted images or memories	2.39 (1.31)	2.05 (1.24)	0.24 (1.25)	** < .001**	0.27
CORE-10 total sum	24.45 (6.12)	18.47 (7.91)	5.97 (6.98)	** < .001**	0.86
** Symptom level for CORE-10 total sum**	**N (%)**	**N (%)**			
Low symptom level (0–10)	19 (2.1)	79 (16.6)			
Mild to moderate symptom level (11–25)	211 (44.4)	281 (59.1)			
Serious symptom level (26–40)	254 (53.5)	115 (24.2)			
**Clinician-reported outcome**					
** Psychiatric and other problems (HoNOS)**	**Mean (SD)**	**Mean (SD)**	**Mean (SD)**	***P***	**Cohen’s d**
1. Overactive, aggressive or agitated	0.39 (0.68)	0.25 (0.54)	0.13 (0.68)	** < .001**	0.10
2. Non-accidental self-injury	0.73 (1.10)	0.33 (0.77)	0.39 (1.10)	** < .001**	0.26
3. Problem drinking or drug-taking	0.44 (0.97)	0.24 (0.68)	0.20 (.073)	** < .001**	0.18
4. Cognitive problems	0.55 (0.74)	0.34 (0.61)	0.21 (0.76)	** < .001**	0.19
5. Physical illness or disability	0.65 (1.11)	0.45 (0.93)	0.20 (0.82)	** < .001**	0.15
6. Hallucinations and delusions	0.17 (0.60)	0.10 (0.44)	0.07 (0.41)	** < .001**	0.08
7. Depressed mood	2.15 (0.98)	1.45 (1.03)	0.71 (1.07)	** < .001**	0.56
8. Other mental and behavior problems	2.61 (0.93)	1.91 (1.02)	0.68 (1.13)	** < .001**	0.50
9. Problems with relationships	1.46 (1.16)	1.18 (1.01)	0.28 (1.10)	** < .001**	0.17
10. Problems in activities of daily living	1.08 (1.10)	0.75 (0.94)	0.33 (1.12)	** < .001**	0.20
11. Problems with living conditions	0.27 (0.79)	0.21 (0.65)	0.06 (0.70)	.055	0.00
12. Problems with occupation and activities	0.78 (1.18)	0.68 (1.00)	0.11 (1.17)	.039	0.01
HoNOS total sum	11.16 (4.84)	6.56 (4.00)	4.59 (4.43)	** < .001**	1.04
				**End of treatment**	
**Patient satisfaction with treatment (CSQ8)**				**Mean (SD)**	
1. How would you rate the quality of the service you have received?				3.56 (0.62)	
2. Did you get the kind of service you wanted?				3.47 (0.61)	
3. To what extent did the program meet your needs?				3.28 (0.68)	
4. If a friend were in need of similar help, would you recommend the program to him or her?				3.71 (0.49)	
5. How satisfied are you with the amount of help you have received?				3.51 (0.68)	
6. Have the services you received help you deal more effectively with your problems?				3.47 (0.58)	
7. In an overall general sense, how satisfied are you with the services you have received?				3.58 (0.58)	
8. If you were to seek help again, would you come back to the service?				3.62 (0.58)	
Service user satisfaction (CSQ8 total sum)				28.19 (3.74)	

*CORE-10* Clinical Outcome in Routine Evaluation 10, *HoNOS* Health of the Nation Outcome Scales, *GSQ-8* Client Satisfaction Questionnaire 8

The clinician-reported outcome (HoNOS) was significantly positive for the total sum and for items 1 to 10, while items 11 and 12 had no significant change. The effect size for the total sum was large, and the effect size for items 1 to 10 was small for eight items and medium for the two items depressed moods and other problems (mostly anxiety).

Data on patient satisfaction (CSQ8) were only collected at the end of the treatment. Most patients were very satisfied, with a mean score approaching 3.5 or higher for all items.

Crisis treatment including use of crisis beds: Altogether, 21 participants were admitted to an inpatient unit as a part of their treatment. Twelve participants spent 137 days altogether in local inpatient units where the CRT was located, and nine participants spent 79 days altogether in a hospital mental health inpatient unit. There were no significant differences between those with and without the use of inpatient stay for patient-reported outcome (CORE-10 change in total sum 7.29 (SD 6.39) vs. 5.91 (SD 7.00), t 0.882, p 0.378), clinician-reported outcome (HoNOS change in total sum 6.43 (SD 5.01) vs. 4.51 (SD 4.39), t 1.950, p 0.052), or patient satisfaction at the end of treatment (CSQ-8 total sum 28.38 (SD 3.83) vs. 28.19 (SD 3.74), t 0.234, p 0.815).

Ways of ending the crisis treatment: At the end of eight weeks of crisis treatment, 35 service users (7.4%) remained in treatment by the CRT; 413 (86.9%) had been discharged from CRT treatment to other services or with no need for further services, and 45 (9.5%) had been admitted to a mental health inpatient unit (34 to an inpatient unit at the local CMHC, ten to a hospital inpatient unit, and one to an inpatient unit for substance abuse). Data on type of discharge from the CRT were missing for 17 service users (3.6%).

### Factors associated with outcome

Table 3 shows the factors significantly associated with patient-reported outcomes (CORE-10) in the linear mixed models. Seven of the 29 independent variables were included in Model AB, with the lowest BIC value. Patient-reported symptoms at admission, medication management, and patient satisfaction were positively associated with patient-reported outcomes. Previously known mental illness, duration of crisis or problems, self-referral, and collaboration with mental health wards were negatively associated with patient-reported outcomes. In Model A, living alone was an additional variable with a negative significant association with patient-reported outcomes. In Model B, additional variables with a positive significant association with patient-reported outcomes were practical support and medication management, and an additional variable with a significant negative association was time to the first meeting after the referral. The results of the complete models for the patient-reported outcomes (CORE-10) with all the independent variables are available in Supplementary Table A in the online supplementary material.

**Table 3:** Association of background and treatment variables to patient reported outcome (CORE-10 pre-post) of treatment by crisis resolution teams (*N* = 475). Linear mixed effects models with regression coefficients (RC) and confidence intervals (CI)

Variables	RC	CI 95% (lower, higher)		*p*
**Model A: Situation at the start of treatment**				
Living alone	-0.19	-0.31	-0.06	**.003**
Previous known mental illness	-0.21	-0.33	-0.09	**.001**
Patient-reported symptoms (CORE-10) at start	0.37	0.27	0.46	** < .001**
Crisis duration	-0.13	-0.21	-0.05	**.002**
- constant	0.37	0.01	0.73	.042
Team level variance^a^: 9.4%				
**Model B: Treatment provided**				
Self-referral	-0.20	-0.36	-0.04	**.014**
Time to first meeting after received referral	-0.06	-0.11	-0.01	**.013**
Treatment duration (weeks)	0.02	0.00	0.04	**.051**
Practical support	0.37	0.06	0.67	**.019**
Medication management	0.29	0.04	0.54	**.024**
Collaboration with mental health inpatient units	-0.37	-0.71	-0.02	**.040**
Patient satisfaction (CSQ-8)	0.47	0.35	0.59	** < .001**
- constant	-0.99	-1.45	-0.53	< .001
Team level variance^a^: 8.1%				
**Model AB: Situation at the start of treatment and treatment provided**				
Previous known mental illness	-0.23	-0.34	-0.12	** < .001**
Patient-reported symptoms (CORE 10) at admission	0.38	0.29	0.47	** < .001**
Crisis duration	-0.10	-0.17	-0.03	**.009**
Self-referral	-0.21	-0.35	-0.06	**.005**
Medication management	0.36	0.13	0.60	**.002**
Collaboration with mental health inpatient units	-0.55	-0.88	-0.23	**.001**
CS Patient satisfaction (CSQ-8)	0.51	0.39	0.63	** < .001**
- constant	-1.60	-2.14	-1.05	< .001
Team level variance^a^: 9.3%				

^a^Intraclass correlation coefficient (ICC) calculated using empty linear mixed models with only intercepts

Table 4 shows the factors significantly associated with the clinician-reported outcomes in the linear mixed models. Seven of the 29 independent variables were kept in Model AB with the lowest BIC value. Clinician-reported problems at the start of treatment, patient satisfaction, and treatment duration were positively associated with clinician-reported outcome. Previous contact with the team, patient-reported symptoms at admission, average meeting duration, and collaboration with mental health wards were negatively associated with clinician-reported outcome. In Model B, additional variables with positive significant association with clinician-reported outcomes were practical support and medication management. The results of the complete models for the clinician-reported outcomes (CORE-10) with all the independent variables are available in Supplementary Table B in the online supplementary material.

**Table 4:** Association of background and treatment variables to clinician reported outcome (HoNOS pre-post) of treatment by crisis resolution teams (*N* = 475). Linear mixed effects models with regression coefficients (RC) and confidence intervals (CI)

Variables	RC	CI 95% (lower, higher)		*p*
**Model A: Situation at the start of treatment**				
Patient-reported symptoms (CORE-10) at start	-0.09	-0.14	-0.03	**.002**
Clinician-reported problems (HoNOS) at start	0.54	0.45	0.62	** < .001**
- constant	-0.01	-0.14	0.12	.843
Team level variance^a^: 17.2%				
**Model B: Treatment provided**				
Treatment duration (weeks)	0.02	0.00	0.03	**.008**
Practical support	0.26	0.07	0.44	**.006**
Medication management	0.19	0.04	0.34	**.012**
Collaboration with mental health inpatient units	-0.21	-0.42	0.00	**.046**
Patient satisfaction (CSQ-8)	0.12	0.04	0.19	**.002**
- constant	-0.23	-0.49	0.03	.082
Team level variance^a^: 12.9%				
**Model AB: Situation at the start of treatment and treatment provided**				
Earlier contact with crisis team	-0.09	-0.15	-0.02	**.013**
Patient-reported symptoms (CORE-10) at start	-0.07	-0.12	-0.02	**.003**
Clinician-reported symptoms (HoNOS) at start	0.59	0.52	0.67	** < .001**
Treatment duration (weeks)	0.02	0.01	0.03	**.002**
Average duration of sessions (ordinal scale)	-0.11	-0.16	-0.05	** < .001**
Collaboration with mental health inpatient units	-0.43	-0.60	-0.25	** < .001**
Patient satisfaction (CSQ-8)	0.14	0.08	0.21	** < .001**
- constant	-0.29	-0.60	0.02	.064
Team level variance^a^: 13.5%				

^a^Intraclass correlation coefficient (ICC) calculated using empty linear mixed models with only intercepts

Table 5 shows the factors significantly associated with patient satisfaction in the linear mixed models. Four of the 29 independent variables were kept in Model AB with the lowest BIC value. Age, sex (female > male), crisis support from family and network, and treatment length were positively associated with patient satisfaction. In Model B, an additional variable with a negative significant association with patient satisfaction was time to first meeting after receiving referral. The results of the complete models for the patient satisfaction (CSQ-8) with all the independent variables are available in Supplementary Table C in the online supplementary material.

**Table 5:** Association of background and treatment variables to patient satisfaction (CSQ-8 post) of treatment by crisis resolution teams (*N* = 475). Linear mixed effects models with regression coefficients (RC) and confidence intervals (CI)

Variables	RC	CI 95% (lower, higher)		*p*
**Model A: Situation at the start of treatment**				
Age group	0.04	0.01	0.06	**.008**
Sex	0.12	0.03	0.20	**.005**
Crisis support (CSS)	0.08	0.03	0.12	** < .001**
- constant	3.02	2.82	3.23	< .001
Team level variance^a^: 6.8%				
**Model B: Treatment provided**				
Time to first meeting after referral	-0.04	-0.08	-0.01	**.023**
Treatment length (weeks)	0.02	0.00	0.04	**.018**
- constant	3.56	3.46	3.67	< .001
Team level variance^a^: 6.3%				
**Model AB: Situation at the start of treatment and treatment provided**				
Age group	0.04	0.01	0.06	**.007**
Sex	0.12	0.03	0.20	**.005**
Crisis support (CSS)	0.08	0.04	0.12	**.000**
Treatment length (weeks)	0.02	0.01	0.04	**.004**
- constant	2.92	2.71	3.14	.000
Team level variance^a^: 9,6%				

^a^Intraclass correlation coefficient (ICC) calculated using empty linear mixed models with only intercepts

The total mean fidelity was not significantly associated with any of the outcomes or patient satisfaction.

### Difference in outcome between teams

As reported in Tables 3, 4 and 5, the portion of variance at team level was 8–9% for patient-reported outcomes, 13–19% for clinician-reported outcomes, and 6–10% for patient satisfaction. According to our definitions presented in the data analysis section, the team-level proportion of the total variance (ICC) was low for patient-reported outcomes and patient satisfaction and medium for clinician-reported outcomes.

In a previous paper, we reported that treatment accessibility was higher for cluster A of teams than for cluster B [18]. Comparing outcomes using independent t-test between cluster A and B of teams showed no significant differences for patient-reported outcome (CORE-10 change 5.65 (SD 7.13) vs. 6.41 (SD 6.75), t -1.137, p 0.256) or patient satisfaction at treatment end (CSQ-8 total sum 28.25 (SD 3.71) vs. 28.10 (SD 3.84), t 0.406, p 0.685). However, cluster A had a significantly higher clinician-reported outcome than cluster B (HoNOS change 4.91 (SD 4.27) vs. 4.03 (SD 4.57), t 2.099, p 0.036).

## Discussion

### Outcomes and patient satisfaction

Patient-reported outcomes significantly improved during crisis treatment, with the most improvement in feelings of hopelessness, unhappiness, and anxiety. Anxiety is often used in various therapies to predict outcomes and adjust treatment. While the distribution of symptom severity among the service users showed a pattern of movement to lower severity, one of four service users still had serious symptoms at treatment end. Our study showed a high level of distress for service users seeking help from CRTs, as well as a large reduction in distress over eight weeks or less. We have not found any other studies using CORE-10 or other patient-reported symptom measures as an outcome measure in CRTs. Given the increasing emphasis on listening to service users in the last decades, it is surprising that few studies on CRTs have assessed patient-reported outcomes. However, several studies on CRT outcomes have used patient satisfaction as an outcome measure [7, 13, 30, 31, 38]. This may reflect that service users have expressed a preference for quality of life and personal recovery as more important than a reduction in symptoms, which has often been the focus of health personnel providing treatment.

The limited use of patient-reported outcome measures may indicate that choices of outcomes in CRT studies have focused on interests within mental health policy and practices. Further, more involvement of service user experts in the choice of research questions and design of studies may lead to more focus on patient-reported outcomes. Recent publications on treatments and health services also focus more on subjective experiences and life conditions that are important for quality of life. One study found that loneliness and social isolation were associated with poorer self-rated recovery following a crisis. This finding indicates a potential role for interventions targeting loneliness and social isolation as important to improving recovery for people with mental health symptoms [39]. An ongoing trial of home treatment in Switzerland includes questionnaires on psychiatric symptoms, well-being, self-efficacy, and emotion regulation to examine potential patient- and relationship-related predictors of treatment success [40].

Clinician-reported outcomes showed significant improvement for the HoNOS total sum as well as for the first ten items. The effect size for the improvement was large for the total sum and small or medium for the first ten HoNOS items on symptoms, behavior, and functioning. The nonsignificant change for items 11 and 12 (problems with living conditions, occupation, and activities) was expected for this patient group during crisis treatment for a few weeks. The HoNOS total sum change pre to post was above the outcome measured in a previous pre-post study of Norwegian CRTs [10] and in line with a UK randomized controlled trial [31] and two recent pre-post studies from Spain [11, 12].

The effect size was large for both the patient-reported outcomes and clinician-reported outcomes in our study. Most of the questions in CORE-10 are about feelings and thoughts, while HoNOS was designed to cover problems of more serious mental illness in several areas of health, behavior, symptoms, and functioning. Used together, these two supplementary measurements may give a broader picture of outcome as reported by service users and clinicians as key informants and cover important areas of outcome.

As patient satisfaction (CSQ-8) could only be assessed at the end of treatment, we have not measured changes in patient satisfaction. However, when comparing our findings with patient satisfaction in other CRT studies, our study was similar to the patient satisfaction (CSQ-8 total sum 28) for both the 15 experimental teams and ten control teams in a cluster-randomized study on the effect of training CRTs in the UK, where there was no significant difference between the two groups of teams at the end of the treatment [9]. This was a similar or higher patient satisfaction than measured as outcome with the same questionnaire in a previous pre-post study and a randomized controlled trial in the UK [30, 31] as well as in a recent CRT study in the Netherlands [6] and a CRT study in Greece using a questionnaire on patient satisfaction developed for the study. Thus, several studies have found that patients value CRTs.

### Factors associated with outcomes

Practical support and medication management had the highest and most consistent positive associations with outcomes for both patient- and clinician-reported outcome. Practical support was among the help service users valued most in a Norwegian qualitative study [41]. These findings may indicate that service users with more serious problems are more likely to need practical support and medication management, and that they are more likely to benefit from CRT treatment when this also includes practical support and medication management.

Collaboration with mental health inpatient units had negative associations with both patient- and clinician-reported outcomes. As describe under Methods, CRTs’ collaboration with inpatient units was mostly preparing and implementing inpatient admissions. Admission to inpatient units may indicate that the service user had more serious mental health problems, limiting the measured outcome of CRT treatment before inpatient admission, and also that the inpatient admission reduced the length of the CRT treatment giving less time for improvement.

We have not found other studies with analyses of the association of patient-reported and clinician-reported outcomes with other factors.

Treatment duration in weeks was positively associated with both outcomes and patient satisfaction, comparable to an earlier study in Norway, which found that length of treatment predicted favorable outcomes [10]. However, the regression coefficient was low for all three associations, indicating that other aspects of treatment may be more important than treatment duration. The average duration of sessions was negatively associated with clinician-reported outcome, perhaps indicating that the clinicians had longer sessions with the service users they found most difficult to help. Patient-reported outcome was negatively associated with previously known mental illness, duration of crisis or problems, and collaboration with mental health wards as a part of the treatment. This may indicate that patients with more serious mental illness and/or longer duration of problems had less improvement and more often needed transfer to inpatient care.

Psychological interventions and family involvement were not included in the final models of factors associated with outcomes. In a Dutch study of patients treated by a crisis and home treatment team, the involvement of relatives was not associated with differences in patient-reported outcome levels of psychiatric symptoms [42]. However, due to limitations in the study, the authors state that this should be studied further with more rigorous methods. Training CRTs in involving family in the treatment may also be helpful [43]. Regarding psychological interventions, there has been little focus on which psychological interventions have been used in CRTs. The scoping review mentioned in the introduction [2] identified only two studies that found promising results of specific psychological interventions: a cognitive behavioral approach (Comprehend, Cope, and Connect) [44] and Eye Movement Desensitization and Reprocessing Therapy [45]. Recent efforts to identify and evaluate psychological interventions in CRTs include surveys among psychologists working with acute patients and the benefits of using a “crisis toolbox” with cognitive techniques [46, 47].

Psychological interventions were provided to almost all service users, and the lack of variation in this variable may explain the lack of an association with outcomes. Of the variables measuring access to the CRT, self-referral and response time from the received referral to the first session had negative associations with the patient-reported outcome. None of the variables on access to the CRT were kept in the final models for clinician-reported outcomes.

Patient-reported duration of the crisis or problems at baseline were negatively associated with the patient-reported outcome. The patient-reported severity of the crisis at baseline was positively associated with patient-reported outcome and negatively associated with the clinician-reported outcome. In comparison, the clinician-reported severity of the service user’s problems at the start of the treatment had a positive association with the clinician-reported outcome and was not included in the final models for the patient-reported outcome. This means each group of informants reported more change when the same informant group had assessed higher problem severity at treatment start. Contributing reasons for this may be a larger possibility for improvement with higher severity of problems at the start of treatment, as well as a regression toward the mean. The negative association of self-referral with patient-reported outcomes is more difficult to understand but could indicate that patients with resources and initiative to contact the CRTs directly may experience fewer problems and need less improvement. One earlier study found that self-referral was not associated with favorable outcomes [10].

Previous mental illness had a negative association with patient-reported outcomes, and earlier contact with the team had a negative association with clinician-reported outcomes. The only variable on patient characteristics in the final models for outcomes was living alone, which had a negative association with patient-reported outcomes. Age, sex, and psychosis were not included in the final models for outcomes.

Patient satisfaction at the end of the treatment was positively associated with age, being female, crisis support from family and network, and treatment length. This indicates that older and female patients were more satisfied with the treatment and that support from family and informal networks, as well as longer treatment, also contributed to higher patient satisfaction.

### Differences in outcome between teams

The linear mixed model analyses showed that some of the total variance of the outcomes was at the team level. In the analyses of the two CRT clusters with higher and lower levels of accessibility, only the clinician-reported outcomes showed a significant difference in outcomes. In our previous article on accessibility and interventions of these CRTs, we reported several significant differences in accessibility (response time to first session, portion of sessions outside CRT location, portion of sessions outside office hours, intensity [sessions per week]) between these two clusters of teams. In contrast, we found no significant difference for any of the six interventions we measured [18]. In the current study, we found few significant associations for variables measuring various components of accessibility, while we found a significant association between outcomes and two interventions (medication management, practical support). This may indicate that the level of team differences found in the linear mixed model analyses of outcomes is more related to differences in interventions than differences in access.

Total fidelity is not included in the final linear mixed models of factors associated with outcomes. One reason for this may be that the difference in fidelity between the teams in the study is not large enough [36]. Another reason may be that total fidelity is the sum of 47 items and that any association between outcomes and some items on key CRT components may be hidden when total fidelity is the variable in the data analysis. As fidelity is a measure at the team level, it may also be possible to find significant associations when we analyze the associations between outcome measured at the individual level and interventions provided at the individual level.

### Strengths and limitations

The sample of patients is large, and the 25 participating CRTs were almost half of the CRTs in Norway and from urban and rural areas in all four health regions. The study used well-established and validated questionnaires and rating scales. A major limitation is that the study had no control group, and we cannot prove to what extent the outcomes in our study are caused by the CRT treatment and to what extent this may be a regression toward the mean over time. Another limitation was that the 25 CRTs in this study signed up voluntarily to participate, so the sample may have had an overrepresentation of teams with more engagement in getting feedback on their practice. CRTs in smaller CMHCs and Northern Norway are underrepresented in the study. Interrater reliability of the fidelity ratings was not calculated, as the fidelity assessors did not do independent ratings of fidelity before agreeing on scores by consensus.

## Conclusions

Both the patient- outcomes and clinician-reported outcomes were significantly positive and with a large effect size. Both were significantly positively associated with practical support and medication management and negatively associated with collaboration with mental health inpatient units. Patient satisfaction at the end of the treatment was high. Some of the variation in outcomes was at the team level. Patient- and clinician-reported outcomes should be used more in studies on the effect of treatment provided by crisis resolution teams.

### Supplementary Information

**Additional file 1: Supplementary Table A.** Association of background and treatment variables to patient-reported outcome (CORE-10 change pre-post) of treatment by crisis resolution teams (*N*=475). Linear mixed effects models with regression coefficients (RC) and confidence intervals (CI). Complete models with all variables included, before reduction of variables. **Supplementary Table B.** Association of background and treatment variables to clinician-reported outcome (HoNOS change pre-post) of treatment by crisis resolution teams (*N*=475). Linear mixed effects models with regression coefficients (RC) and confidence intervals (CI). Complete models with all variables included, before reduction of variables. **Supplementary Table C.** Association of background and treatment variables to patient satisfaction (CSQ-8 at the end of treatment) of treatment by crisis resolution teams (*N*=475). Linear mixed effects models with regression coefficients (RC) and confidence intervals (CI). Complete models with all variables included, before reduction of variables.

## Data Availability

The dataset used and analyzed during the current study is available from the corresponding author on reasonable request.

## Abbreviations

CMHC: Community mental health center
CORE-10: Clinical Outcome in Routine Evaluation 10
CRT: Crisis resolution team
CSS: Crisis Support Scale
CSAS: Crisis State Assessment Scale
CSQ-8: Client Satisfaction Questionnaire 8
GP: General practitioner
HoNOS: Health of the Nation Outcome Scales
ICC: Intraclass correlation coefficients
