# Supplementary material for: Patient-reported outcome, clinician-reported outcome, and patient satisfaction with treatment by crisis resolution teams: a multicenter pre-post study of outcome and associated factors in Norway
Source: BMC Psychiatry. 2024 Jan 31;24:82. doi: 10.1186/s12888-024-05543-3 (PMC10829386; doi:10.1186/s12888-024-05543-3)
Supplement: Supplementary file 1 — Additional file 1: Supplementary Table A. Association of background and treatment variables to patient-reported outcome (CORE-10 change pre-post) of treatment by crisis resolution teams (N=475). Linear mixed effects models with regression coefficients (RC) and confidence intervals (CI). Complete models with all variables included, before reduction of variables. Supplementary Table B. Association of background and treatment variables to clinician-reported outcome (HoNOS change pre-post) of treatment by crisis resolution teams (N=475). Linear mixed effects models with regression coefficients (RC) and confidence intervals (CI). Complete models with all variables included, before reduction of variables. Supplementary Table C. Association of background and treatment variables to patient satisfaction (CSQ-8 at the end of treatment) of treatment by crisis resolution teams (N=475). Linear mixed effects models with regression coefficients (RC) and confidence intervals (CI). Complete models with all variables included, before reduction of variables. [file 12888_2024_5543_MOESM1_ESM.docx]

Online Supplementary Material

Ruud et al. Patient-reported outcome, clinician-reported outcome, and patient satisfaction with treatment by crisis resolution teams: A multicenter pre-post study of outcome and associated factors in Norway. BMC Psychiatry 2024.

**Supplementary Table A.**

**Association of background and treatment variables to patient-reported outcome (CORE-10 change pre-post) of treatment by crisis resolution teams (N=475). Linear mixed effects models with regression coefficients (RC) and confidence intervals (CI). Complete models with all variables included, before reduction of variables**.

| **Model A: Situation at the start of treatment** |  |  |  |  |
| --- | --- | --- | --- | --- |
| **Variables** | **RC** | **CI 95% (lower, higher)** | | **p** |
| Age group | .0146408 | -.0243502 | .0536318 | .462 |
| Sex | .0406806 | -.0788898 | .1602511 | .505 |
| Living alone | -.175643 | -.301391 | -.0498949 | .006 |
| Previously known mental illness | -.2030458 | -.3257566 | -.080335 | .001 |
| Psychosis | .0771586 | -.1442098 | .298527 | .495 |
| Earlier contact with the crisis team | .0077261 | -.1335044 | .1489565 | .915 |
| Patient-reported symptoms (CORE-10) at start | .4562159 | .3233269 | .5891049 | <.001 |
| Clinician-reported problems (HoNOS) at start | .0109064 | -.1455285 | .1673414 | .891 |
| Crisis how acute | -.0532992 | -.1126538 | .0060553 | .078 |
| Crisis duration | -.0985893 | -.1837549 | -.0134236 | .023 |
| Crisis support | .0217298 | -.0404399 | .0838994 | .493 |
| Crisis experience (CSAS) at start | -.0103141 | -.1253496 | .1047214 | .861 |
| Not coping with crisis (CSAS) at start | -.0854815 | -.2003997 | .0294367 | .145 |
| - constant | .3834413 | -.1812655 | .9481481 | .183 |
|  |  |  |  |  |
| **Model B: Treatment provided** |  |  |  |  |
| **Variables** | **RC** | **CI 95% (lower, higher)** | | **p** |
| Self-referral | -.1892837 | -.3484443 | -.0301232 | .020 |
| Time to first meeting after referral | -.0812376 | -.1600548 | -.0024203 | .043 |
| Seen on referral day | -.0717954 | -.2541608 | .1105699 | .440 |
| Treatment length (weeks) | .0221704 | -.0009362 | .0452771 | .060 |
| Treatment intensity (sessions per week) | .0256908 | -.0631905 | .114572 | .571 |
| Average duration of sessions (ordinal scale) | -.0736487 | -.1914182 | .0441208 | .220 |
| Proportions of sessions outside team’s location | -.0444285 | -.1888302 | .0999731 | .546 |
| Proportions of sessions outside working hours | -.0888972 | -.297599 | .1198045 | .404 |
| Practical support | .4034489 | .0831779 | .7237199 | .014 |
| Psychological interventions | .0627424 | -.2269499 | .3524346 | .671 |
| Family involvement | .0882762 | -.1572697 | .3338221 | .481 |
| Medication management | .2660326 | .0101344 | .5219308 | .042 |
| Collaboration with mental health inpatient units | -.3446179 | -.6963678 | .007132 | .055 |
| Collaboration with GPs and primary care | -.1036638 | -.3396418 | .1323141 | .389 |
| Patient satisfaction with treatment (SCQ-8) | .4662339 | .3413969 | .5910709 | <.001 |
| - constant | -.7488704 | -1.445948 | -.0517926 | .035 |
|  |  |  |  |  |

| **Model AB: Situation at start of treatment, and treatment provided** |  |  |  |  |
| --- | --- | --- | --- | --- |
| **Variables** | **RC** | **CI 95% (lower, higher)** | | **P** |
| Age group | -.002346 | -.038924 | .034232 | .900 |
| Sex | -.0121316 | -.1238155 | .0995522 | .831 |
| Living alone | -.0916276 | -.2109781 | .0277229 | .132 |
| Previously known mental illness | -.2140298 | -.3266339 | -.1014257 | <.001 |
| Psychosis | .0190129 | -.1870331 | .2250589 | .856 |
| Earlier contact with the crisis team | .0082049 | -.1258301 | .1422399 | .905 |
| Patient-reported symptoms (CORE-10) at start | .4716138 | .3493151 | .5939125 | <.001 |
| Clinician-reported problems (HoNOS) at start | -.0018718 | -.1538078 | .1500643 | .981 |
| Crisis how acute | -.0488187 | -.10353 | .0058926 | .080 |
| Crisis duration | -.0758864 | -.1568268 | .005054 | .066 |
| Crisis support | -.0015049 | -.0596583 | .0566486 | .960 |
| Crisis experience (CSAS) at start | -.048007 | -.155165 | .0591509 | .380 |
| Not coping with crisis (CSAS) at start | -.0624258 | -.1675564 | .0427048 | .245 |
| Self-referral | -.1812476 | -.32755 | -.0349453 | .015 |
| Time to first meeting after referral | -.0237402 | -.0980078 | .0505274 | .531 |
| Seen on referral day | -.0154738 | -.1850528 | .1541052 | .858 |
| Treatment length (weeks) | .0246714 | .0034279 | .0459149 | .023 |
| Treatment intensity (sessions per week) | -.0048594 | -.0892955 | .0795767 | .910 |
| Average duration of sessions (ordinal scale) | -.0407138 | -.1544471 | .0730194 | .483 |
| Proportions of sessions outside team’s location | -.0121409 | -.150462 | .1261801 | .863 |
| Proportions of sessions outside working hours | -.0814325 | -.2696431 | .1067781 | .396 |
| Practical support | .3480111 | .0479468 | .6480755 | .023 |
| Psychological interventions | -.0008486 | -.2706638 | .2689665 | .995 |
| Family involvement | .1363304 | -.0961071 | .3687679 | .250 |
| Medication management | .3472994 | .1060946 | .5885042 | .005 |
| Collaboration with mental health inpatient units | -.4109762 | -.744644 | -.0773085 | .016 |
| Collaboration with GPs and primary care | -.1816894 | -.4027414 | .0393626 | .107 |
| Patient satisfaction with treatment (SCQ-8) | .4715504 | .3528572 | .5902435 | <.001 |
| - constant | **-1.041493** | -1.838611 | -.2443755 | .010 |

**Supplementary Table B.**

**Association of background and treatment variables to clinician-reported outcome (HoNOS change pre-post) of treatment by crisis resolution teams (N=475). Linear mixed effects models with regression coefficients (RC) and confidence intervals (CI). Complete models with all variables included, before reduction of variables**.

| **Model A: Situation at the start of treatment** |  |  |  |  |
| --- | --- | --- | --- | --- |
| **Variables** | **RC** | **CI 95% (lower, higher)** | | **p** |
| Age group | -.0011106 | -.0216471 | .0194259 | .916 |
| Sex | -.0070633 | -.0700832 | .0559566 | .826 |
| Living alone | -.0478077 | -.114038 | .0184226 | .157 |
| Previously known mental illness | .029452 | -.0350345 | .0939385 | .371 |
| Psychosis | .0401684 | -.0765408 | .1568777 | .500 |
| Earlier contact with the crisis team | -.0854891 | -.1597778 | -.0112005 | .024 |
| Patient-reported symptoms (CORE-10) at start | -.0773531 | -.1473431 | -.0073631 | .030 |
| Clinician-reported problems (HoNOS) at start | .5377486 | .4554657 | .6200315 | <.001 |
| Crisis how acute | -.0082258 | -.0395233 | .0230717 | .606 |
| Crisis duration | -.0051813 | -.0500756 | .039713 | .821 |
| Crisis support | .0033393 | -.0294268 | .0361053 | .842 |
| Crisis experience (CSAS) at start | -.0331975 | -.0938018 | .0274068 | .283 |
| Not coping with crisis (CSAS) at start | .0253529 | -.0352143 | .0859201 | .412 |
| _constant | .0776799 | -.219509 | .3748688 | .608 |
|  |  |  |  |  |
| **Model B: Treatment provided** |  |  |  |  |
| **Variables** | **RC** | **CI 95% (lower, higher)** | | **p** |
| Self-referral | -.0643796 | -.1552783 | .0265192 | .165 |
| Time to first meeting after referral | .0101954 | -.0368911 | .0572818 | .671 |
| Seen on referral day | .0813417 | -.0275734 | .1902567 | .143 |
| Treatment length (weeks) | .0194075 | .005589 | .033226 | .006 |
| Treatment intensity (sessions per week) | .0183733 | -.0340347 | .0707812 | .492 |
| Average duration of sessions (ordinal scale) | -.0436598 | -.114565 | .0272454 | .227 |
| Proportions of sessions outside team’s location | .0101473 | -.0757489 | .0960434 | .817 |
| Proportions of sessions outside working hours | -.0163519 | -.1367569 | .1040531 | .790 |
| Practical support | .2509388 | .0603105 | .441567 | .010 |
| Psychological interventions | -.0865873 | -.2598247 | .0866501 | .327 |
| Family involvement | .0546801 | -.0923072 | .2016675 | .466 |
| Medication management | .1325832 | -.0204274 | .2855939 | .089 |
| Collaboration with mental health inpatient units | -.2190941 | -.4301004 | -.0080879 | .042 |
| Collaboration with GPs and primary care | .0072795 | -.1323403 | .1468992 | .919 |
| Patient satisfaction with treatment (SCQ-8) | .1124469 | .0378032 | .1870905 | .003 |
| - constant | -.0695442 | -.4859515 | .346863 | .743 |
|  |  |  |  |  |

| **Model AB: Situation at start of treatment, and treatment provided** |  |  |  |  |
| --- | --- | --- | --- | --- |
| **Variables** | **RC** | **CI 95% (lower, higher)** | | **p** |
| Age group | -.002356 | -.0219711 | .0172592 | .814 |
| Sex | -.0145409 | -.074825 | .0457432 | .636 |
| Living alone | -.0079025 | -.0722473 | .0564423 | .810 |
| Previously known mental illness | .0154245 | -.0452447 | .0760938 | .618 |
| Psychosis | .0208495 | -.0890852 | .1307842 | .710 |
| Earlier contact with the crisis team | -.0841885 | -.1565288 | -.0118481 | .023 |
| Patient-reported symptoms (CORE-10) at start | -.0863702 | -.151993 | -.0207473 | .010 |
| Clinician-reported problems (HoNOS) at start | .5918943 | .5105893 | .6731993 | <.001 |
| Crisis how acute | -.006232 | -.0356119 | .023148 | .678 |
| Crisis duration | .0110504 | -.0324321 | .054533 | .618 |
| Crisis support | .0027296 | -.028633 | .0340923 | .865 |
| Crisis experience (CSAS) at start | -.0300058 | -.0874962 | .0274845 | .306 |
| Not coping with crisis (CSAS) at start | .0384316 | -.0181334 | .0949965 | .183 |
| Self-referral | -.0476383 | -.1239971 | .0287206 | .221 |
| Seen on referral day | .0304945 | -.0302817 | .0912706 | .325 |
| Treatment length (weeks) | .0168417 | .0054787 | .0282047 | .004 |
| Treatment intensity (sessions per week) | .0088639 | -.0358865 | .0536144 | .698 |
| Average duration of sessions (ordinal scale) | -.0838849 | -.1442913 | -.0234786 | .006 |
| Proportions of sessions outside team’s location | -.0125989 | -.0864862 | .0612883 | .738 |
| Proportions of sessions outside working hours | -.0322395 | -.1314886 | .0670097 | .524 |
| Practical support | .0513788 | -.109909 | .2126667 | .532 |
| Psychological interventions | -.02548 | -.1696023 | .1186423 | .729 |
| Family involvement | -.0352083 | -.1599239 | .0895073 | .580 |
| Collaboration with mental health inpatient units | -.4107057 | -.5906515 | -.2307598 | <.001 |
| Collaboration with GPs and primary care | -.1161819 | -.234694 | .0023302 | .055 |
| Patient satisfaction with treatment (SCQ-8) | .1467268 | .0828556 | .2105979 | <.001 |
| - constant | -.3325541 | -.7394335 | .0743252 | .109 |
|  |  |  |  |  |

**Supplementary Table C**

**Association of background and treatment variables to patient satisfaction (CSQ-8 at the end of treatment) of treatment by crisis resolution teams (N=475). Linear mixed effects models with regression coefficients (RC) and confidence intervals (CI). Complete models with all variables included, before reduction of variables**.

| **Model A: Situation at the start of treatment** |  |  |  |  |
| --- | --- | --- | --- | --- |
| **Variables** | **RC** | **CI 95% (lower, higher)** | | **p** |
| Age group | .0361883 | .0089747 | .0634019 | .009 |
| Sex | .1247094 | .0412091 | .2082097 | .003 |
| Living alone | -.0293308 | -.1170647 | .0584031 | .512 |
| Previously known mental illness | -.0311155 | -.1168177 | .0545867 | .477 |
| Psychosis | .0380018 | -.1163546 | .1923582 | .629 |
| Earlier contact with the crisis team | .0937683 | -.0047066 | .1922431 | .062 |
| Patient-reported symptoms (CORE-10) at start | .0037454 | -.088846 | .0963369 | .937 |
| Clinician-reported problems (HoNOS) at start | .0220012 | -.0872183 | .1312207 | .693 |
| Crisis how acute | -.0128239 | -.0542678 | .02862 | .544 |
| Crisis duration | -.0459058 | -.1053833 | .0135716 | .130 |
| Crisis support | .0698335 | .026465 | .113202 | .002 |
| Crisis experience (CSAS) at start | .0749825 | -.005317 | .1552819 | .067 |
| Not coping with crisis (CSAS) at start | -.0455837 | -.1257469 | .0345794 | .265 |
| - constant | 3.06503 | 2.670638 | 3.459422 | <.001 |
|  |  |  |  |  |
| **Model B: Treatment provided** |  |  |  |  |
| **Variables** | **RC** | **CI 95% (lower, higher)** | | **p** |
| Self-referral | .1093741 | -.0033447 | .222093 | .057 |
| Time to first meeting after referral | -.0290247 | -.0857207 | .0276712 | .316 |
| Seen on referral day | .0245467 | -.1067711 | .1558644 | .714 |
| Treatment length (weeks) | .020029 | .0034933 | .0365647 | .018 |
| Treatment intensity (sessions per week) | -.0201679 | -.0840437 | .0437079 | .536 |
| Average duration of sessions (ordinal scale) | -.0265499 | -.1112373 | .0581376 | .539 |
| Proportions of sessions outside team’s location | .1014675 | -.0015946 | .2045296 | .054 |
| Proportions of sessions outside working hours | -.0657966 | -.21427 | .0826768 | .385 |
| Practical support | .1384327 | -.0915726 | .3684379 | .238 |
| Psychological interventions | -.0748848 | -.2833792 | .1336096 | .481 |
| Family involvement | .0788442 | -.097747 | .2554354 | .382 |
| Medication management | .044364 | -.1395334 | .2282614 | .636 |
| Collaboration with mental health inpatient units | .1849032 | -.0681259 | .4379324 | .152 |
| Collaboration with GPs and primary care | -.1721531 | -.3407323 | -.0035738 | .045 |
| - constant | 3.643457 | 3.26321 | 4.023705 | <.001 |
|  |  |  |  |  |

| **Model AB: Situation at start of treatment, and treatment provided** |  |  |  |  |
| --- | --- | --- | --- | --- |
| **Variables** | **RC** | **CI 95% (lower, higher)** | | **p** |
| Age group | .030159 | .002856 | .0574621 | .030 |
| Sex | .117145 | .0338568 | .2004332 | .006 |
| Living alone | -.0143141 | -.1043671 | .0757388 | .755 |
| Previously known mental illness | -.0355696 | -.1205807 | .0494416 | .412 |
| Psychosis | .0278919 | -.1278145 | .1835983 | .726 |
| Earlier contact with the crisis team | .0625705 | -.0386524 | .1637935 | .226 |
| Crisis how acute | -.0182356 | -.0595115 | .0230402 | .387 |
| Crisis duration | -.038953 | -.0998279 | .0219219 | .210 |
| Crisis support | .0739145 | .0312008 | .1166282 | .001 |
| Crisis experience (CSAS) at start | .0691493 | -.0095365 | .1478351 | .085 |
| Not coping with crisis (CSAS) at start | -.0386271 | -.1103803 | .033126 | .291 |
| Self-referral | .1022341 | -.0106186 | .2150869 | .076 |
| Time to first meeting after referral | -.0190769 | -.0750349 | .0368811 | .504 |
| Seen on referral day | .0297656 | -.0981646 | .1576958 | .648 |
| Treatment length (weeks) | .0221427 | .0060715 | .038214 | .007 |
| Treatment intensity (sessions per week) | -.0155835 | -.0798478 | .0486808 | .635 |
| Average duration of sessions (ordinal scale) | -.0314852 | -.1174825 | .0545121 | .473 |
| Proportions of sessions outside team’s location | .0722264 | -.0326394 | .1770923 | .177 |
| Proportions of sessions outside working hours | -.0604334 | -.2056426 | .0847758 | .415 |
| Practical support | .1561604 | -.0678826 | .3802034 | .172 |
| Psychological interventions | -.0194565 | -.2232036 | .1842907 | .852 |
| Family involvement | .0392074 | -.1349483 | .2133631 | .659 |
| Medication management | .0542843 | -.1279874 | .2365559 | .559 |
| Collaboration with mental health inpatient units | .1614752 | -.0876386 | .4105891 | .204 |
| Collaboration with GPs and primary care | -.1731974 | -.3379068 | -.008488 | .039 |
| - constant | 3.14258 | 2.614102 | 3.671058 | <.001 |
